# A Robotics-Based Behavioral Paradigm to Measure Anxiety-Related Responses in Zebrafish

**DOI:** 10.1371/journal.pone.0069661

**Published:** 2013-07-29

**Authors:** Valentina Cianca, Tiziana Bartolini, Maurizio Porfiri, Simone Macrì

**Affiliations:** 1 Department of Mechanical and Aerospace Engineering, Polytechnic Institute of New York University, Brooklyn, New York, United States of America; 2 Section of Behavioural Neuroscience, Department of Cell Biology and Neuroscience, Istituto Superiore di Sanità, Roma, Italy; University Zürich, Switzerland

## Abstract

Zebrafish are gaining momentum as a laboratory animal species for the study of anxiety-related disorders in translational research, whereby they serve a fundamental complement to laboratory rodents. Several anxiety-related behavioral paradigms, which rest upon the presentation of live predatorial stimuli, may yield inconsistent results due to fatigue, habituation, or idiosyncratic responses exhibited by the stimulus itself. To overcome these limitations, we designed and manufactured a fully controllable robot inspired by a natural aquatic predator (Indian leaf fish, *Nandus nandus*) of zebrafish. We report that this robot elicits aversive antipredatorial reactions in a preference test and that data obtained therein correlate with data observed in traditional anxiety- and fear-related tests (light/dark preference and shelter-seeking). Finally, ethanol administration (0.25; 0.50; 1.00%) exerts anxiolytic effects, thus supporting the view that robotic stimuli can be used in the analysis of anxiety-related behaviors in zebrafish.

## Introduction

Behavioral phenotyping in animal models of psychiatric disorders is historically challenged with a series of requirements: replacement of laboratory mammals with animals characterized by a lower central nervous system development (partial replacement); reduction of the number of animals used; and refinement of housing and testing procedures (three R's principle) [1]. Within this principle, it is necessary to devise testing procedures that maximize the output of a given experiment while minimizing animal use, suffering, and neuroanatomical complexity.

Zebrafish have recently emerged as a relevant experimental species for the investigation of functional and dysfunctional biological processes due to the sequencing of their genome, their high reproduction rate, their short intergeneration time, and their elevated stocking density compared to laboratory mammals [2]–[5]. Kalueff and collaborators [6] have recently epitomized the relevance of zebrafish as a valid experimental tool to investigate the biological determinants of emotions. Thus, zebrafish have been used to investigate the fundamental mechanisms governing individual response to drugs of abuse [7]–[12], exhibition of emotional patterns [13]–[17], and higher order brain functions, such as learning and memory [18]–[21]. Specifically, several classical studies adopted zebrafish to study the genetic determinants of drug addiction [22], [23] and personality [24]. In light of such growing interest, several behavioral paradigms have been developed [8], [12], [13], [25]. Some of these efforts leveraged the adoption of fully automated test strategies [26], [27].

With respect to the analysis of emotional responses, a broad spectrum of experimental approaches have been directly translated into zebrafish research from traditional tests originally developed for rodents [28]. The different paradigms adopted to investigate anxiety- and fear-related reactions attempted to signal the presence of a predator through several modalities, ranging from chemosensory stimuli to visual cues. Chemosensory stimuli have been successfully used to elicit alarm reactions in zebrafish [29], [30]. While this approach has resulted in practical and theoretical advancements, it does not constitute an ideal scenario whereby olfactory cues are difficult to experimentally handle [3]. Different studies adopted the innate aversion for darkened environments and allowed zebrafish to freely explore bi-partitioned compartments [13], [14], [25], [31]–[36].

Beside reiterating the preference of zebrafish for bright areas, these studies allowed the analysis of anxiety-related behaviors in response to anxiogenic and anxiolytic drugs. Specifically, zebrafish preference for the light compartment has been shown to vary in response to the administration of drugs conventionally used to influence anxiety states in humans [32], [36], [37]. Yet, the reliability of this test is uncertain, whereby the preference for the bright compartment has been shown to fluctuate across different studies [33], [38]. The presence of a lid over the dark compartment has been shown to constitute the main factor capable of influencing zebrafish preference for the light vs. dark compartment [38]. Thus, while in the absence of a cover, zebrafish generally prefer the dark compartment, the inclusion of a lid induces a preference for the light compartment. Specifically, the presence of a lid in the darkened portion of the apparatus offers a “cave-like” stimulus, which live zebrafish would naturally avoid.

Likewise, resting upon the natural aversion of this freshwater species to predatorial stimuli, experimental subjects have been confronted with visual stimuli, either constituted by sympatric and allopatric predators or generated through computer animations [39]–[41]. Specifically, zebrafish show a fear response in the presence of both the Indian leaf fish (*Nandus nandus*) [39] and a bird silhouette moved on the side or above the tank [40], [41]. The success of these paradigms depends on the characteristic of the zebrafish to navigate in and interact with their environment primarily through their highly functional visual system. While the use of live stimuli allows for a direct induction of fear states, it permits only minimal flexibility for controlling and dissecting specific predatorial features. Additionally, live stimuli can display inconsistent behavioral patterns due to natural physiological fluctuations and therefore they may not constitute entirely controllable independent variables. Such features are readily controllable in computer animations; however, computerized images may fail in reproducing the complexity of live predators [42].

**Figure 1:**
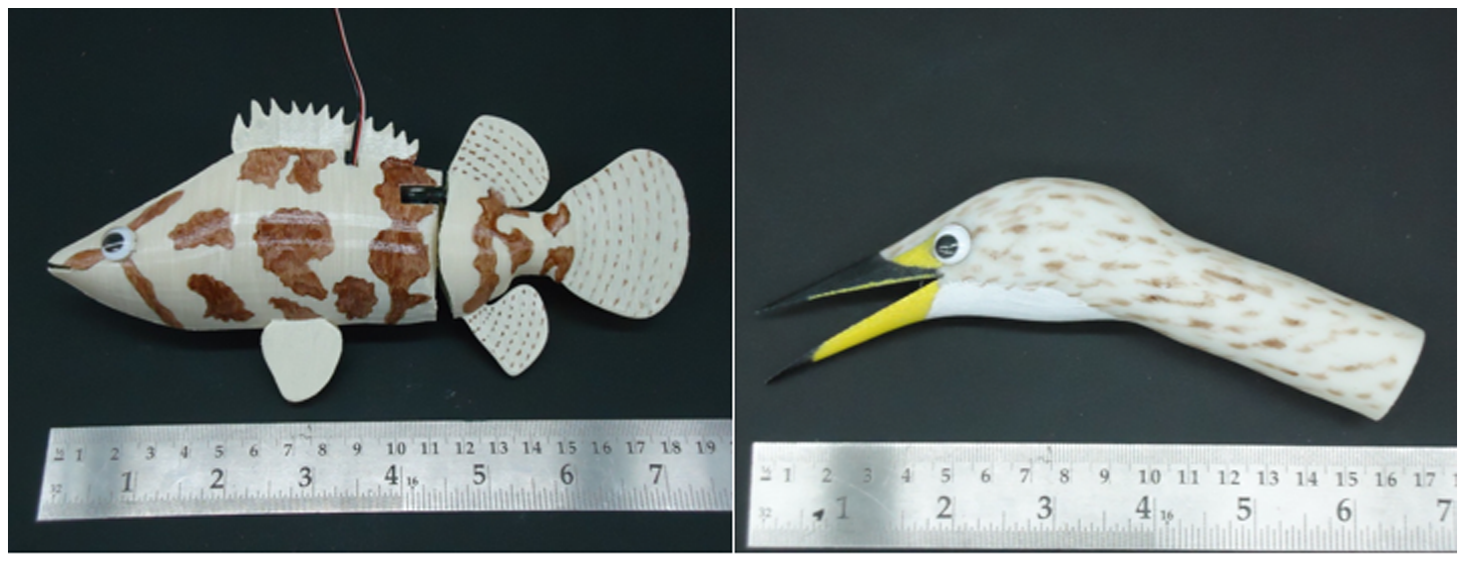
Illustration of the robotic fish (left) and heron (right) used in the robot avoidance and shelter-seeking tests.

Bioinspired robotics may constitute a novel approach capable of bridging these gaps (controllability and complexity) while maximizing the benefits described above [43]. In this framework, we recently designed and manufactured a class of robotic stimuli capable of influencing zebrafish behavior in a series of preference experiments [44]–[46]. These studies have shown that bioinspired robotics constitute a valuable tool in the modulation of zebrafish behavior, by demonstrating that individual subjects and small shoals can be attracted to a robotic fish inspired by zebrafish in its color, shape, and motility. Here, we propose a novel robotic stimulus to serve as a methodological tool for the analysis of fear and anxiety-related emotional responses in zebrafish. Specifically, we designed a robot mimicking the morphology and locomotion pattern of the Indian leaf fish, a natural aquatic predator, and measured the responses it elicited in an avoidance test. This Indian leaf fish-like robot (see Fig. 1a) was used in a novel avoidance test in which live zebrafish were allowed to swim in a tank partitioned into three regions comprising a central part, where the experimental fish were introduced, and two opposite sides juxtaposing robot with an empty compartment. We then compared experimental data obtained in this test with data obtained in two traditional anxiety- and fear-evoking experimental paradigms [13], [25], [31]–[35]: a light/dark (L/D) preference test [14], [33] and a shelter-seeking test in response to the simulation of an attack performed by a heron (Fig. 1b) impacting the water surface [47]. With respect to the latter, we measured the latency of zebrafish to access a sheltered area.

To evaluate whether drugs involved in the modulation of anxiety and fear also regulate individual response in these tests, we exposed separate groups of fish to acute treatments with different doses of ethanol. Although it exerts a plethora of effects, ethanol has been shown to influence anxiety-related responses in a dose-dependent manner across a wide spectrum of experimental paradigms [12], [32], [37].

Ultimately, to support the hypothesis that bioinspired robots may be used in the analysis of fear and anxiety-related states in zebrafish, the following predictions were expected to be met: (1) live zebrafish should display avoidance reactions to a robot designed to resemble a live aquatic predator; (2) individual performance in the aforementioned test should correlate with the traditional anxiety tests (L/D and shelter-seeking test); and (3) exposure to psychoactive compounds affecting anxiety-related traits in humans should modify individual behavior in these tests. To test these predictions, we exposed 120 zebrafish to these three tests under baseline conditions or in response to three doses of ethanol.

## Materials and Methods

### Ethics Statement

All animal handling and experimental procedures were performed according to European Communities guidelines (EC Council Directive 86/609), Italian legislation on animal experimentation (Decreto L.vo 116/92) and NIH guide for the care and use of laboratory animals. The experiment described in this work was approved by the Polytechnic Institute of New York University (NYU-Poly) Animal Welfare Oversight Committee AWOC 2012-101 and AWOC 2013-103.

### Animals and housing

A total of 184 wild-type zebrafish (*Danio rerio*) were obtained from an online aquaria source (LiveAquaria.com, Rhinelande, Wisconsin, USA). All fish were given at least two weeks to acclimate to the laboratory environment and were kept in a large holding tank (76.5 cm×31.5 cm×47.5 cm). All fish were housed with a density of 1.06 fish/liter during the acclimatization period. Zebrafish involved in this study were considered young adult sexually mature, according to their main body length, which was about three cm total length. Animals were fed daily with commercial flake food (tropical fish flakes formula specifically prepared by Petland Discount, Brooklyn, for cyprinid species) after the conclusion of the daily experimental session. Illumination was provided by full spectrum fluorescent light for ten hours each day, according to circadian rhythm of this species. Water was maintained at 26±1°C and pH at 7.2. All fish used in this study were experimentally naïve and, in order to avoid psudo-randomization, they were used only once.

### Apparatuses

In the first series of experiments, each subject was tested sequentially in three test paradigms (diagrams of the setups used in the present study and videos of the tests involving robotic stimuli are reported in the supporting information): a L/D preference test (see Figure S1), a robotics-based predator avoidance test (see Figure S2 and Video S1) involving a robotic stimulus that resembles the Indian leaf fish (see Fig. 1a), and a shelter-seeking experiment (see Figure S3) involving a prototype mimicking an Indian pond heron (*Ardeola grayii*) (see Fig. 1b and Video S2). In order to visually isolate experimental subjects from the testing room, test tanks were surrounded by opaque curtains. In the second series of experiments, each subject was tested in one of the following conditions: still robotic fish (tail beat frequency = 0.0 Hz); fast robotic fish (tail beat frequency = 0.4 Hz); beige robotic fish (tail beat frequency = 0.2 Hz); and brown robotic fish (tail beat frequency = 0.2 Hz).

#### Light/dark preference test

The apparatus consisted of a small tank (30.5 cm×15 cm×20.5 cm) divided in two equally sized compartments with varying light conditions. The walls of the apparatus were rendered white and black with the use of white and black cardboard paper, respectively. The tank was selectively illuminated from underneath using white (36 inches fluorescent aquarium reflector, 20 Watt, All-Glass Aquarium, INC Franklin Wisconsin) and infrared lights (Wisecomm, Infrared LED night light, China) for the light and dark compartments, respectively. Specifically, the bottom of the dark compartment was covered with a black photographic filter (Lee Filters, Andover, England) allowing infrared lights to pass through [15]. A transparent plastic filter of equivalent size and thickness was used for the light compartment. In addition, a white plastic panel was placed under the tank to diffuse white light. Light and filter positioning resulted in a marked separation between the light and dark compartment: specifically, the illuminance was respectively 7 and 1 lux. A physical barrier was not placed between the two compartments and no lid was placed above them. An infrared camera (Creative Labs, VF0205, China) was located at 49 cm from the water surface of the experimental setup to score fish position in both compartments.

#### Robotics-based predator avoidance test

The apparatus consisted of a rectangular tank (74 cm×30 cm×32 cm) partitioned into three sections. The two side compartments were 10 cm long and the central region was 54 cm long. The compartments were physically separated by two transparent Plexiglas solid panels. The tank was illuminated by two fluorescent lights (36 inches deluxe fluorescent aquarium reflector, 38 Watt, All-Glass Aquarium, INC Franklin Wisconsin) placed along the top tank lateral borders to obtain homogenous illumination of the environment. A webcam (Logitech Webcam Pro 9000) was mounted 65 cm above the water surface for recording fish behavior. A robotic fish was positioned in one of the two compartments, while the other compartment remained empty.

The robotic fish was made of rigid acrylonitrile butadiene styrene (ABS) plastic body shell and tail section. The robot was modelled in SolidWorks and printed on a rapid prototyping machine (Stratasys, Dimension SST, USA), see Figure 1a. Its design was inspired by the Indian leaf fish (*Nandus nandus*) a sympatric predator that evokes anti-predatory responses in zebrafish. In order to develop the robotic stimulus we used the “International Union for Conservation of Nature and Natural Resources” (http://www.iucnredlist.org/apps/redlist/details/166429/0)_ENREF_54 as a reference (19 cm long, 9 cm high, and 3 cm wide). The robot utilized a servomotor (Traxxas, 2065 Sub-Micro servomotor, USA) for actuating the tail controlled by an external microcontroller (Arduino, Duemilanove, Italy). The visual appearance of the robotic fish was obtained by spray-painting the robot with an ivory base color followed by the hand painting of brown color patterns typical for this species, as well as the attachment of small plastic eyes. The microcontroller was connected to a power supply for uninterrupted operation and allows for the selection of two parameters: the tail beating frequency and amplitude. The robot was anchored to the experimental tank with a small metallic support-rod and the same rod was placed in the empty compartment to balance the visual background for the live subjects. The 24.5 cm long rods had diameter of 0.5 cm. The tail beat frequency of the robotic fish was 0.2 Hz and was selected in order to mimic an Indian leaf fish standing still for predation. The tail beat amplitude was 5 cm peak-to-peak. The robot was placed at the bottom of the tank oriented along the short side of the tank.

In order to control for the effect of the color, we used two replicas of the robot inspired by the Indian leaf fish that were painted either with the same ivory color used as base for the Indian fish robot (beige robotic fish) or with the same brown color (brown robotic fish) used to paint the patterns typical of this species (see Figures S4 and S5). To control the motility effect, we varied the tail beat frequency in two additional experiments: specifically we adopted tail beat frequencies of 0 and 0.4 Hz, respectively.

#### Shelter-seeking test

The experimental tank, illumination, and recording system used for this experiment were the same used for the robotics-based predator avoidance test described above. Differently than the latter, in this experiment, the tank was not partitioned into compartments, while an opaque Plexiglas panel was placed over one side of the tank to cover a length of 20 cm to be used by fish as a shelter. In the central part of the tank, a heron head apparatus was installed next to the tank. A slit was made in the curtain surrounding the tank to allow the heron head to remain hidden prior to and immediately after striking the water.

A model of heron (*Ardeola grayii*) was selected as an artificial predator of the fish, since this species was a native predator of zebrafish [40]. The stimulus was designed to simulate the heron quickly striking the surface of the water from above in hunting-type behavior. To achieve this effect, a 32 cm long steel rod was capped with a plastic heron head, designed in SolidWorks and fabricated using the same rapid prototyping machine as for the robotic fish, see Fig. 1b. The bird head had an approximate length of 17 cm and approximate diameter of 2.6 cm. The head and rod were attached to a servomotor (Hitec, SubMicroServoU, USA) capable of pivoting 90 degrees between positions perpendicular and parallel to the water surface. The stimulus was designed to maintain an upright position, move quickly to the parallel position in a striking motion, and then recover to the upright position. The motion of the arm was controlled through a microcontroller (Arduino, Duemilanove, Italy).

### Drugs and treatments

To evaluate the consequences of acute ethanol (EtOH) exposure, we designed a study involving four experimental groups (30 subjects each). Subjects were treated with EtOH at different doses (EtOH 0.00%, EtOH 0.25%, EtOH 0.50%, EtOH 1.00%, where EtOH % represents the corresponding alcohol concentration measured in volume percentage). For EtOH administration, 60 minutes prior to behavioral testing, the subject was placed into a 500 ml plastic beaker filled with water from the holding tank in which the corresponding alcohol dose was dissolved. This immersion period is known to be sufficient to achieve maximal blood and brain alcohol levels [12]. Specifically, brain ethanol concentrations following this treatment schedule are reported to be approximately 90% of those present in the beaker [48]. EtOH concentration in the experimental tank was identical to the concentration in the beaker.

This administration procedure has been reported not to lead to mortality or to lasting physiological changes [12] and has been used in several studies addressing the short-term behavioral effects of alcohol administration in adult zebrafish [32], [48], [49]. Furthermore, Kily and collaborators [50] suggested that this ethanol regime results in brain ethanol concentrations of approximately 40 mmol l^−1^. Additionally, the highest concentration used in the present study has been reported not to affect visual acuity [51]. Fish were always manually transferred between tanks and beaker using a hand net.

### Experimental design and procedure

Tests were divided in two sessions; the first session (entailing the L/D, the shelter seeking and the robotics-based predator avoidance test with a single robot swimming at 0.2 Hz) was conducted in May and June 2012 and the second one (robotics-based predator avoidance test with robotic replicas exhibiting variable pigmentation or tail beat frequency) was conducted in April 2013. All experiments were performed from Monday to Saturday, between 10 am and 6 pm.

In the first session, the L/D preference test was always executed at first since it was conducted in a separate tank, different from the tank used for the remaining two tests, which were systematically alternated within each group. This testing strategy was aimed at minimizing animal disturbance. For each of the three tests, the spatial location of the stimuli (left *versus* right) was systematically varied and the temporal distribution of experiments (morning *versus* afternoon) was counterbalanced across experimental subjects within each group. Behavioral observations for each subject lasted approximately 50 minutes. The L/D preference test consisted of 30 s habituation followed by five minutes of experimental observation. After completion of the test, the subject was placed for three minutes in the beaker filled with the corresponding ethanol dosage. Fish were then placed in the second experimental tank, where they were tested either in the robotics-based predator avoidance test or in the shelter-seeking experiment. Experimental sessions were equal to five and ten minutes for robotics-based predator avoidance and shelter-seeking experiment, respectively. After the test was completed, the subject was placed again in the beaker for approximately three minutes, and then released in the experimental tank to complete the remaining test.

For the shelter-seeking test, at the end of a 10-min habituation period when the experimental fish was outside the shelter area, the heron head was moved such as to simulate an “attack” by striking the water 20 cm from the shelter. Fish that remained underneath the shelter area for the entire test session were excluded from the analysis. Beginning at the deployment of the simulated attack, fish activity was recorded for 10 minutes using the webcam and the latency to access the shelter was recorded.

In order to address whether robot avoidance was dependent on motility or pigmentation, we performed a second series of experiments in which we varied the color of the robot or its tail beat frequency (see above and Figures S4 and S5); in this case, we only performed the robotics-based predator avoidance test. Thus, a fish was placed in a beaker 50 minutes before the test performance to reproduce the same condition of behavioral observations that in the first session lasted approximately 50 minutes each. The test session was equal to fifteen minutes divided in three 5-min bins, out of which we scored the first and the last. The spatial location of the stimuli were counterbalanced across experimental subjects.

### Behavioral observations

Data acquired through a camera were saved on a personal computer for offline analysis. Concerning the L/D test, the following spatio-temporal and ethological parameters were scored: percent time spent in the dark compartment and number of transitions between the light and the dark compartments. The ethological parameters considered were the following: “swimming” (locomotion in any direction), “freezing” (motionless state during which only the gills and the eyes may move), and “thrashing” (moving back and forth against the tank glass while physically in contact with the glass) [45]. For the robotics-based predator avoidance test, the compartment in which the fish was allowed to swim was virtually subdivided into three equally sized sections of 18 cm each: empty side, central part, and stimulus side. The behavioral measures were identical to those measured in the L/D test. Finally, for the shelter-seeking test, we scored the latency to enter the shelter area following the presentation of the threatening stimulus [47]. Data for the L/D test and for the robotics-based predator avoidance test were scored through the Observer 2.0 (Noldus, Wageningen, the Netherlands). Data from the shelter-seeking test were scored through a stop-watch.

### Data analysis

In the preference tests (L/D and robotics-based predator avoidance), we first analyzed whether groups showed a preference for one or the other side of the apparatus and then whether preference varied across experimental groups. These analyses were performed through Chi-square tests followed by non-parametric post-hoc tests using Bonferroni correction. Specifically, we divided the significant p value, p<0.05, by the six independent comparisons; this ultimately resulted in a significant p value, for p<0.05, of p<0.008. We then analyzed the presence or absence of preference for one or the other side of the test tank through Chi-square tests performed within each experimental group. The measures collected in the L/D and in the robotics-based predator avoidance test were analyzed through repeated-measures ANOVA for split-plot designs. The general model was a 4 (treatment)×2 (time-bin) design: treatment was a between subject factor and time-bin was a within subject factor. The general model for the analysis of the ethological parameters was a 4 (treatment)×2 (time-bin)×3 (compartments) design. For the analysis of the shelter-seeking test only the factor treatment (4 levels) was included in the statistical design. The significance level was set at p<0.05. Fisher's protected least significant difference (PLSD) post-hoc tests were used when a significant main effect of the condition variable was observed.

## Results

All experimental raw data have been condensed in an excel file and made freely available for further analyses (see Table S1).

### Light/dark preference test

**Figure 2:**
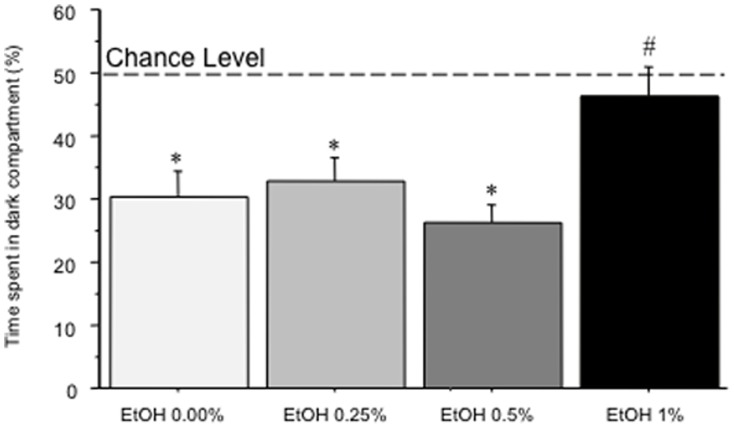
Percent time (mean+SEM) spent in the dark compartment of the L/D test by EtOH 0.00%, EtOH 0.25%, EtOH 0.50%, and EtOH 1.00%. * p<0.05 significant preference for the bright compartment; # p<0.05 significantly different from EtOH in post-hoc comparisons.

As expected, experimental subjects showed a general aversion for the dark side of the apparatus (χ^2^(1) = 38.5, p<0.01); yet, such aversion differed across the four experimental groups (χ^2^(3) = 22.3, p<0.01); specifically, while control (EtOH 0.00%), EtOH 0.25%, and EtOH 0.50% subjects showed a clear aversion for the dark compartment of the test tank (p<0.01, see Fig. 2), EtOH 1.00% individuals failed to show any preference for one or the other side of the apparatus (p = 0.273). Furthermore, EtOH 1.00% zebrafish spent a significantly longer amount of time in the dark compartment compared to the other groups (main effect of treatment: F(3,116) = 4.99, p<0.01; p<0.01 in post hoc tests).

Ethanol administration also modified the time budgeting of experimental subjects with respect to swimming (main effect of treatment: F(3,116) = 5.30, p<0.01), thrashing (main effect of treatment: F(3,116) = 3.24, p<0.05), and freezing (main effect of treatment: F(3,116) = 5.70, p<0.01). With respect to swimming, in the absence of significant differences between EtOH 0.00%, EtOH 0.25%, and EtOH 0.50% subjects, EtOH 1.00% zebrafish showed a significant reduction compared to controls (see Table 1 for values and statistical significance). Complementarily, EtOH 1.00% zebrafish showed a significant increase in the time spent freezing compared to the other groups (see Table 1). Finally, EtOH 0.00% controls showed a significant increase in thrashing behavior compared to the other groups (Table 1).

**Table 1:** Behaviors exhibited in the L/D and robotics-based predator avoidance test.

	EtOH 0.00%	EtOH 0.25%	EtOH 0.50%	EtOH 1.00%	F		p
	Mean ± SEM	Mean ± SEM	Mean ± SEM	Mean ± SEM	DF		
L/D preference Test							
Swimming	265.05±11.57	256.07±16.48	276.53±10.33	201.53±19.66*	3	4.92	0.003
Thrashing	3.08±1.449	0.62±0.223	0.66±0.333	0.63±0.256	3	2.56	0.058
Freezing	31.86±11.743	43.30±16.536	22.81±10.382	97.84±19.753*	3	4.98	0.003
Robot avoidance Test							
Swimming	275.34±9.635	266.97±16.730	275.83±11.284	171.15±21.496*	3	10.89	<0.001
Thrashing	3.27±1.268	1.31±0.491	2.05±0.718	0.53±0.335	3	2.19	0.093
Freezing	21.39±9.785	31.72±16.811	22.12±11.356	128.32±21.601*	3	11.14	<0.0001

Behavioral ethogram exhibited by the four experimental groups in the L/D and robotics-based predator avoidance test. In the two rightmost columns, we report the F and p values observed in the general ANOVA.

* p<0.05 significantly different from EtOH in post-hoc comparisons.

### Robotics-based predator avoidance test

**Figure 3:**
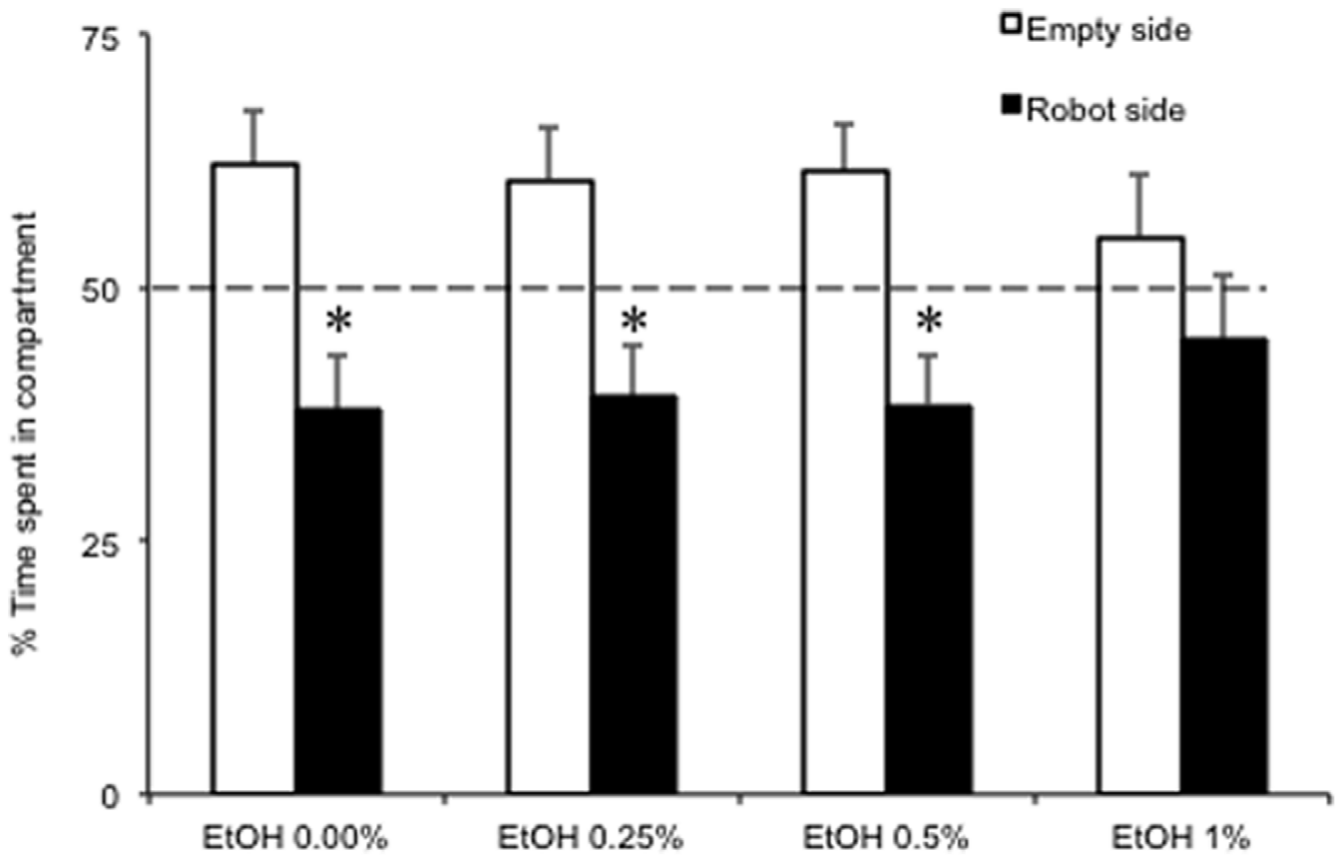
Percent time (mean+SEM) spent near the robot and the empty compartments in the robot avoidance test by EtOH 0.00%, EtOH 0.25%, EtOH 0.50%, and EtOH 1.00%. * p<0.05 significant preference for the empty compartment. Note that the percent time is computed by excluding the time spent in the central part. The dashed line indicates the chance level.

As expected, the Indian leaf fish-like robot elicited aversion in the experimental groups. Thus, experimental subjects showed, on average, a preference for the empty compartment compared to the stimulus compartment ((χ^2^(1) = 8.2, p<0.05, see Fig. 3). Yet, such preference varied across the four experimental groups (χ^2^(3) = 22.3, p<0.05). Thus, control subjects showed a greater avoidance of the robot compared to EtOH 1.00% subjects (p<0.05 in post-hoc tests) which, in turn, failed to show any preference for one or the other side of the apparatus (p = 0.273). As also observed in the L/D test, ethanol consumption altered the time budgeting of the experimental subjects. Specifically, compared to the other groups, EtOH 1.00% subjects showed reduced swimming and thrashing behavior (main effect of treatment: F(3,115) = 13.40, p<0.01 and F(3,115) = 3.88, p<0.05, respectively, see Table 1) and significantly increased freezing (main effect of treatment: F(3,115) = 14.02, p<0.01).

### Shelter-seeking test

**Figure 4:**
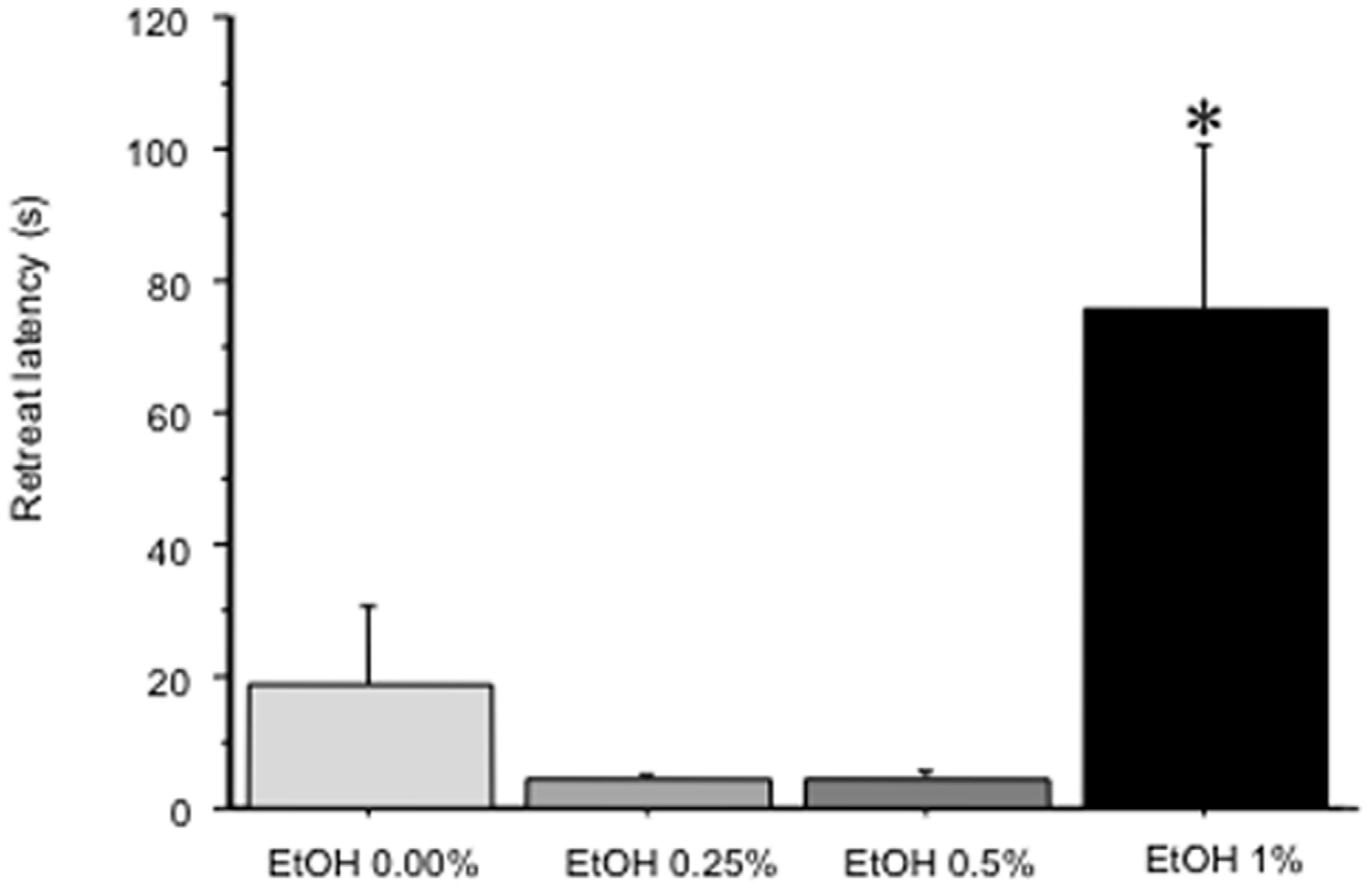
Mean (+SEM) latency (s) to retreat to the shelter following the simulated attack of the heron prototype exhibited by EtOH 0.00%, EtOH 0.25%, EtOH 0.50%, and EtOH 1.00%. * p<0.05 significantly different from EtOH 0.00%.

All of the experimental subjects retreated to the shelter within a short latency following the predatorial attack. Yet, such latency varied depending on the experimental group: thus, EtOH 1.00% subjects showed a longer latency compared to the other groups (F(3,80) = 6.53, p<0.01, p<0.05 in post-hoc tests; see Fig. 4).

### Robotics-based predator avoidance tests with variable color and tail beat frequency

Experimental subjects displayed a remarkable avoidance of moving robots, regardless of their tail beat frequency. Thus, during the 5-min sessions, all subjects showed on average a marked preference for the empty side when confronted with the reference robot beating its tail at 0.4 Hz (χ^2^(1) = 11.267, p<0.001; time spent in the robot side = 59.0±17.6 s and time spent in the empty side = 166.3±15.6 s), with the beige robot (χ^2^(1) = 12.250, p<0.001; time spent in the robot side = 55.5±15.4 s and time spent in the empty side = 184.6±16.2 s), and with the brown robot (χ^2^(1) = 12.250, p<0.001; time spent in the robot side = 60.7±12.1 s and time spent in the empty side = 159.8±13.3 s). Conversely, when confronted with the still robot, subjects failed to show any preference for one or the other side of the apparatus (χ^2^(1) = 0.600, p = 0.439; time spent in the robot side = 147.5±21.8 s and time spent in the empty side = 94.6±13.8 s). Additionally, preference data remained constant throughout the entire experimental session and were not modulated by habituation effects (condition×time bins F(3,58) = 2.110, p = 0.108).

### Correlational studies

**Figure 5:**
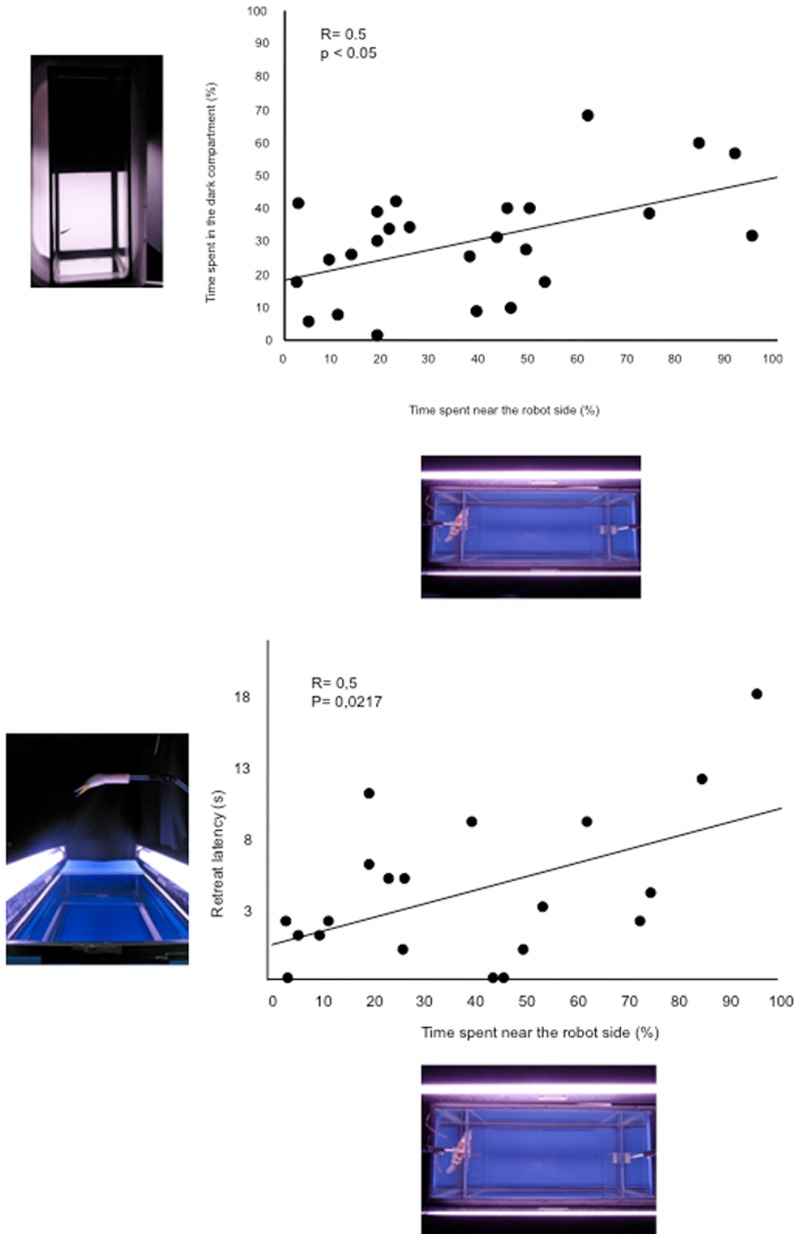
Correlation between the time spent in the dark compartment of the L/D test and the time spent near the robot side in the robot avoidance test (upper panel). Correlation between the latency to retreat to the shelter in the shelter-seeking test and the time spent near the robot side in the robot avoidance test (lower panel).

In order to evaluate whether experimental data obtained in the in the robot-avoidance test were potential indicators of anxiety-related responses, we correlated them with data obtained in the other tests. Specifically, we performed – within the control group – a series of correlations between data observed in the robot avoidance and in the L/D and shelter-seeking test respectively (see Fig. 5a,b). As expected, we observed that the time spent by control subjects in proximity of the robot positively correlated with the time spent in the dark section of the L/D test (R = 0.5 p<0.05) and with the latency to enter the shelter following the presentation of the predatorial stimulus (R = 0.5 p<0.05).

Ultimately, to investigate whether ethanol-dependent reductions in swimming explained the results observed in the shelter-seeking test, we performed independent correlations between the time spent swimming in the L/D test and the latency to retreat to the shelter. These correlations were performed, separately, in all experimental groups. While the correlation between time spent swimming and retreat latency was not significant in EtOH 0.00% (R = 0.055, p = 0.817), EtOH 0.25% (R = 0.236, p = 0.289), and EtOH 0.50% (R = 0.187, p = 0.405), it was significant in EtOH 1.00% (R = 0.545, p = 0.016).

## Discussion

The use of bioinspired robots to regulate live animal behavior may inform the design of novel test strategies, whereby the morphology, motility, and behavioral patterns of such robots can be fully controlled. This aspect is particularly relevant in scenarios in which the independent variable is constituted by a social stimulus. Specifically, situations in which experimental subjects are confronted with socially relevant stimuli generally involve the use of live animals as independent variables (be the latter conspecifics or predators). Independent variables should be fully controllable throughout the entire experiment and should not be influenced by experimental subjects. These assumptions are at risk when using live stimuli as independent variables, as they can exhibit behavioral variations idiosyncratic to the specific test conditions. Aspects like inter-individual interactions, tiredness, and circadian rhythms can remarkably influence their behavior. We believe that robotics may partly fill these gaps and complement traditional test strategies whereby it allows the use of fully controllable independent social variables. In the present study, we evaluated whether robotic stimuli can be used as independent variables in anxiety-related tests in zebrafish. To achieve this goal, we adopted two complementary approaches: on the one hand we evaluated spontaneous aversion towards the robot in control subjects and compared data observed therein with data collected in traditional anxiety tests (L/D and shelter-seeking tests); on the other hand we performed a preliminary pharmacological validation in which we evaluated whether fish behavior varied in response to the administration of ethanol, a psychoactive compound capable of modulating anxiety in humans [52], [53], rodents [54] and fish [32], [41].

As expected, live zebrafish showed a marked aversion for the predatorial robot (resembling the morphology and motility of the natural zebrafish predator *Nandus nandus*); such aversion significantly correlated with the preference for the bright compartment in the L/D test and with the latency to access the protected area in the shelter-seeking test. Ethanol exposure remarkably modulated fish behavior in all three tests. Specifically, zebrafish exposed to an elevated ethanol concentration failed to display predator aversion, preference for the bright compartment and, compared to control individuals, showed a much longer latency to access the shelter in response to the predatorial attack. Ethanol administration, *per se*, significantly modified individual fish behavioral time budgeting whereby it increased freezing and reduced swimming time.

### Ethological validation of the robotics-based predator avoidance test

Robust experimental evidence demonstrate that zebrafish exhibit predictable reactions when confronted with aversive stimuli in captive conditions [55], [56]. For example, Bass and Gerlai [57] demonstrated that zebrafish exposed to predatorial stimuli of variable nature – e.g. live predators or static images projected on a computer screen – exhibit sudden escape reactions (see also [55]). Among the different tests aimed at evaluating fear- and anxiety-related parameters in zebrafish, the L/D test has received robust experimental support. Specifically, several studies demonstrated that, in a L/D test, zebrafish display a clear preference for the bright compartment [25] and that such preference can be modulated with anxiolytic drugs, including ethanol [38]. In line with previous literature, we observed that zebrafish exhibit a marked preference for the bright compartment. However, we note that, in contrast with our observations, several studies reported that zebrafish exhibit a remarkable baseline preference for the dark instead of the light compartment [14], [33]. Champagne and collaborators [25] systematically addressed the factors potentially influencing individual preference for one or the other compartment. Specifically, they observed that the presence of a lid covering the dark compartment shifted individual preference from the dark to the bright side of the apparatus. Although we did not include a lid in the present setup, we positioned light sources to maximize the difference in illumination between the two compartments; additionally, we adopted cardboard paper to cover the external walls of the tank and photographic filters to further darken the bottom of the tank. We thus offer that our setup was similar to that used by Gerlai and collaborators [32] and by Champagne et al [25]; using this setup, both groups exhibited a significant preference for the light versus the dark compartment.

The second test we adopted in the present study was a simulation of an aerial attack performed by a heron prototype impacting the water surface when fish were swimming in the unprotected sector of the experimental tank. This test has been used in several aquatic species, including sea bass and trout and has been demonstrated to be sensitive to treatments capable of modulating anxiety [47], [58]. This test does not feature an internal control whereby it does not involve an approach-avoidance conflict (that is, internal drive to explore the entire apparatus counterbalanced by the potential danger associated with the dark and with the robot containing compartments). Yet, it can reliably be used to further corroborate the data obtained in the robot avoidance experiment and to evaluate individual responses to the administration of psychoactive compounds (ethanol in the present study). As expected, we observed that when exposed to the aerial attack, control fish readily retreated to the shelter area.

The findings observed in the L/D and in the shelter-seeking tests served as the cornerstone against which testing the feasibility of using robotic stimuli as novel fear-based experimental tasks. Specifically, we anticipated that performance in a novel robotics-based anxiety test should correlate with traditional tests mapping onto the same psychobiological construct. While correlational analyses may prove useful in highlighting the interdependency between different parameters, in the present study they may entail some intrinsic limitations. Specifically, in order to validate our robotic-based test, we needed to assess zebrafish in a sequential test battery. While we counterbalanced the presentation of the predator avoidance test and the shelter-seeking test to overcome test-battery effects, we always performed the L/D test first due to technical constraints. Individual response to this test may thus have influenced individual response in the novel paradigms, thereby potentially influencing correlational data.

In keeping with our predictions, zebrafish showed a clear aversion for the robot; such aversion significantly correlated with the performance exhibited in the other tests. We believe that the aversion for the robot depends on its biomimetic characteristics. Specifically, the robot imitates *Nandus nandus* across a series of features including its shape, color, dimensions (see Fig. 1), and tail beat frequency. To further detail the aspects that influenced individual avoidance of the robot, we performed a series of experiments in which we systematically varied the robot color and tail beat frequency. In these studies we observed that tail beating dictates individual aversion towards the robot. Specifically, live zebrafish exhibited a clear aversion towards any moving Indian leaf-shaped robot regardless of color or pigmentation. Conversely, in the absence of tail beating, live zebrafish spent approximately 50% of their time in proximity of the robotic stimulus. Furthermore, the avoidance of the swimming robot does not seem to be influenced by habituation to the stimulus. Specifically, the set of experiments involving systematically variable robots entailed a 15-min long experimental session. Such length allowed for the analysis of the temporal pattern of individual exploration. This analysis demonstrated that robot avoidance was elevated throughout the session and did neither increase nor dissipate over time.

Beside the observation that fish display a clear aversion for the robot, the significant correlation between data observed in this test and in the L/D test supports the view that robots may be used in anxiety-related choice paradigms. Specifically, fish that exhibited the highest aversion for the robot also spent the longest amount of time in the bright section of the L/D test. Additionally, we observed that the escape latency significantly correlated with the time spent in the proximity of the robot. Thus, fish that spent longer amounts of time in proximity of the robotic aquatic predator also took a longer latency to retreat to the shelter.

### Pharmacological validation

In order to further assess the suitability of our approach, we attempted to pharmacologically validate it through the use of a compound with known anxiolytic properties. To this aim, we evaluated whether individual responses to the robotic stimulus were sensitive to the administration of ethanol. Although ethanol is not a pure anxiolytic compound, it has been extensively used in zebrafish to validate novel anxiety-related tests [32], [38], [48], [59], [60]. The suitability of ethanol as a compound against which testing the validity of novel experimental paradigms rests on its easy manageability, rapid diffusion in body fluids and extensive use in several laboratory animal species (including mice [61], rats [54], and fish [32]).

In line with our predictions, ethanol administration reduced anxiety-like behaviors in all the tests performed. Specifically, the administration of an elevated dose of ethanol abolished individual aversion for the robot, for the dark side of the L/D test and increased the latency to retreat to the shelter following the aerial attack. Yet, ethanol administration also remarkably influenced individual time budgeting in terms of swimming (reduction) and freezing (increment). With respect to the latter, two aspects warrant further discussion: on the one hand, freezing is generally considered an indicator of anxiety [40], [41] and, therefore, increased freezing may equate to increased anxiety; on the other hand, since the experimental paradigms used in the present study heavily rest on locomotion, it may be argued that ethanol-dependent variations in these tasks depend on alterations in general motility rather than on anxiety-related factors. With respect to the first argument, we suggest that ethanol-induced freezing is unlikely to represent an anxiety-like response. Rather, we believe that it depends on generalized effects of ethanol on absolute levels of locomotion. Thus, ethanol has been shown to reduce locomotion, independently of the potentially threatening nature of the stimulus, in many different species including rodents, zebrafish, and humans [62], [63] through a direct action at the level of midbrain dopaminergic neurons [61]. With respect to the possibility that reduced locomotion may explain the results observed in the tests adopted in the present study, we offer that two of these protocols rest upon respective preference and not upon general locomotion. Thus, experimental fish may decide to spend time in proximity of one or the other stimulus – regardless of the specific behavioral pattern exhibited – and locomotion should not significantly interfere with such choice. Therefore, we believe that ethanol-induced variations in the time spent in the dark compartment of the L/D test and in the proximity of the robot in the avoidance test may reflect variations in the anxiety domain rather than being explained by alterations in absolute levels of locomotion.

Yet, impaired locomotion may partly explain the results observed in the shelter-seeking test: this proposition is further supported by the observation that, in EtOH 1.00% subjects, the time spent swimming in the L/D significantly correlated with the escape latency observed in the shelter-seeking test. Future studies are needed to evaluate whether classical anxiolytic compounds, without major side effects on locomotion, increase the latency to retreat to the shelter.

### Conclusions and future perspectives

In the present study, we showed that robotics can be used to elicit fear reactions in zebrafish and that this tool can be utilized to evaluate emotional responses in this emerging laboratory animal species. Thus, this study offers novel avenues to partially replace – in anxiety-related tests – laboratory mammals with animal species characterized by a lower neurological complexity. The possibility to employ robots as independent variables substantially increases the controllability of experimental conditions, thus potentially favoring the reproducibility of experimental findings [64]. Additionally, in the present study, we adopted a psychoactive compound (ethanol) that, besides modulating anxiety, also impinges on different psychobiological domains (e.g. general locomotion and reward systems). Future studies are needed to evaluate whether the platform proposed herein is sensitive to “pure” anxiolytics. Ultimately, future studies should evaluate whether robotic stimuli may influence anxiety-related behavior not only in isolated individuals but also in small groups of subjects. This research avenue may also inform several related disciplines, ranging from the study of collective behavior to animal protection, production, and control [44], [65].

## Supporting Information

Figure S1 **graphical sketch of the setup used for the Light/dark preference test.**
(PDF)

Figure S2 **graphical sketch of the setup used for the Robotics-based predator avoidance test.**
(PDF)

Figure S3 **graphical sketch of the setup used for the Shelter-seeking test.**
(PDF)

Figure S4 **Illustration of the beige robot used in the control experiments investigating the effect of pigmentation.**
(JPG)

Figure S5 **Illustration of the brown robot used in the control experiments investigating the effect of pigmentation.**
(JPG)

Table S1 **Experimental raw data.**
(XLSX)

Video S1 **Robot: representative recording of the interaction between a live fish and the biologically-inspired robot resembling an Indian leaf fish (Nandus nandus).**
(MOV)

Video S2 **Heron: representative recording of the reaction exhibited by live fish to the heron prototype impacting the water surface.**
(MOV)
