# Supplementary figures and images for: A Robotics-Based Behavioral Paradigm to Measure Anxiety-Related Responses in Zebrafish
Source: PLoS One. 2013 Jul 29;8(7):e69661. doi: 10.1371/journal.pone.0069661 (PMC3726767; doi:10.1371/journal.pone.0069661)

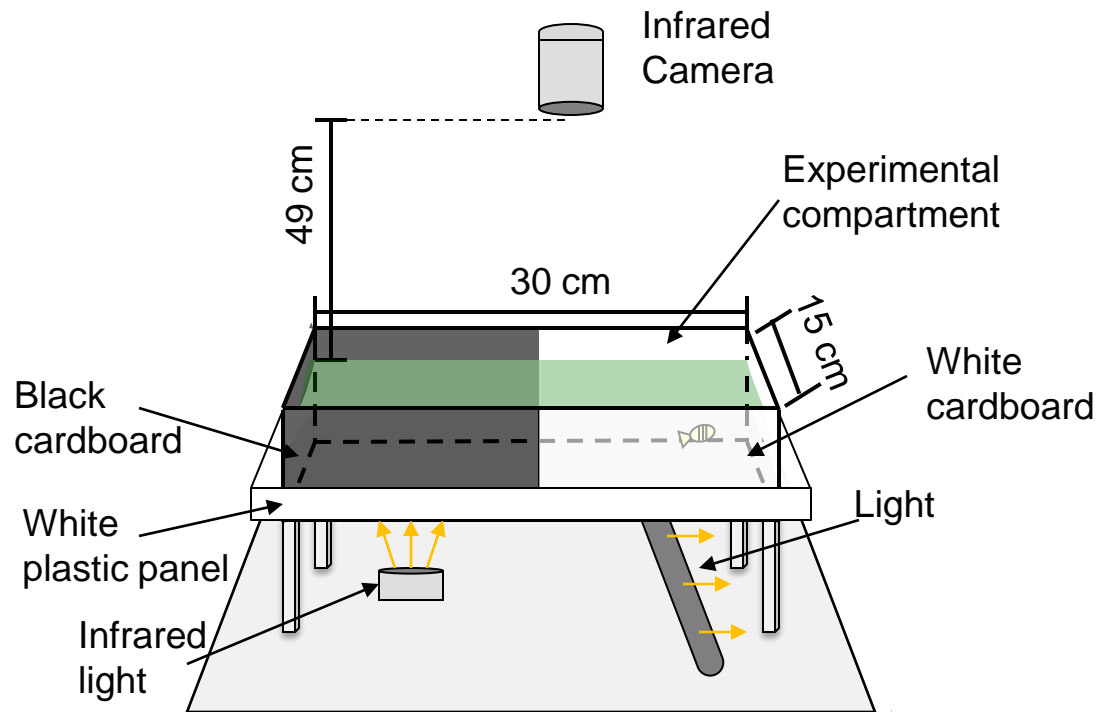

Supplement: Figure S1 — graphical sketch of the setup used for the Light/dark preference test. (PDF) [file pone.0069661.s001.pdf]

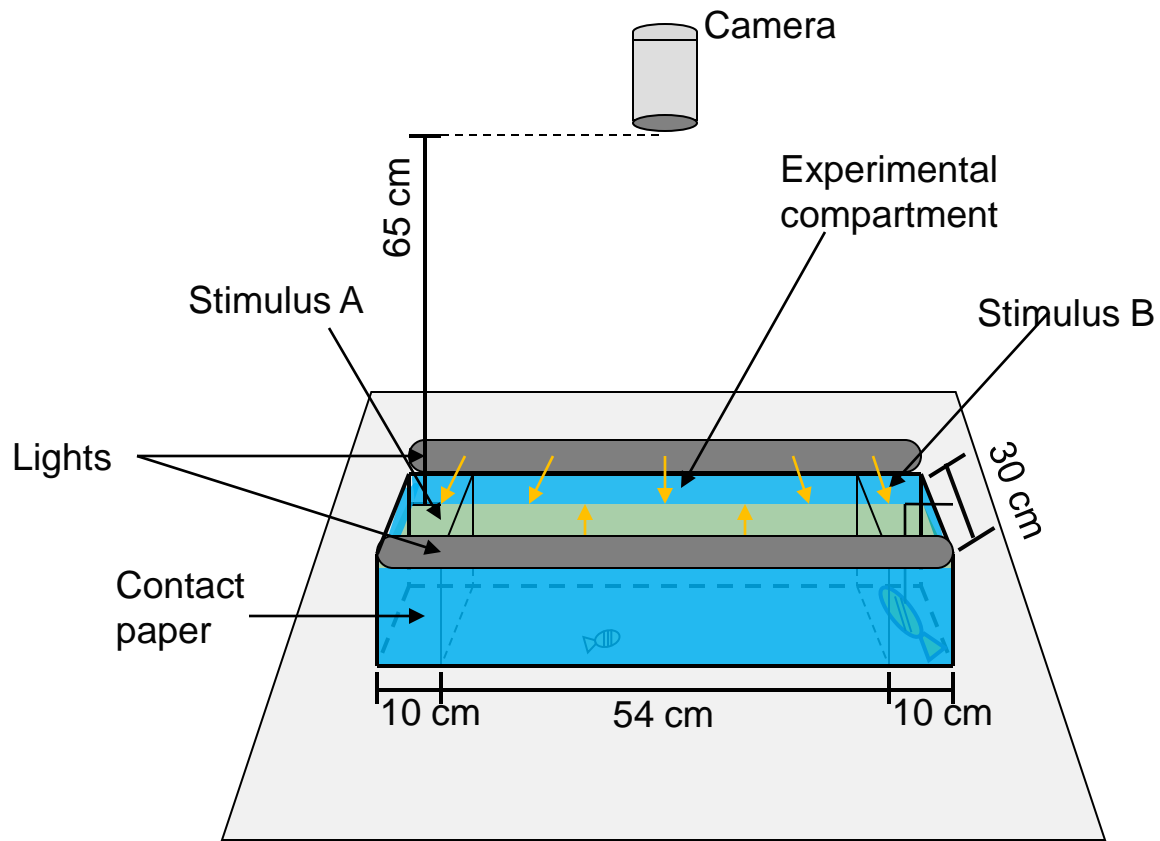

Supplement: Figure S2 — graphical sketch of the setup used for the Robotics-based predator avoidance test. (PDF) [file pone.0069661.s002.pdf]

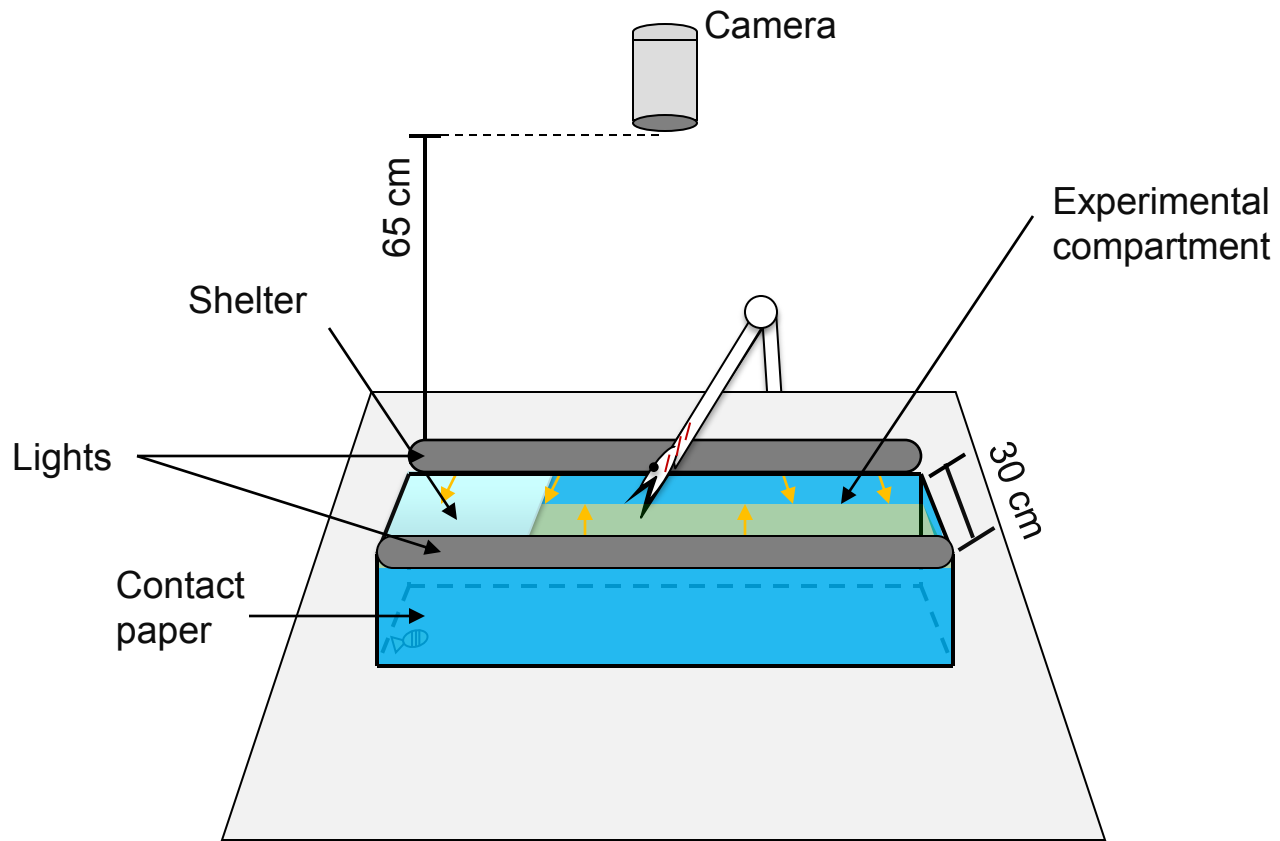

Supplement: Figure S3 — graphical sketch of the setup used for the Shelter-seeking test. (PDF) [file pone.0069661.s003.pdf]

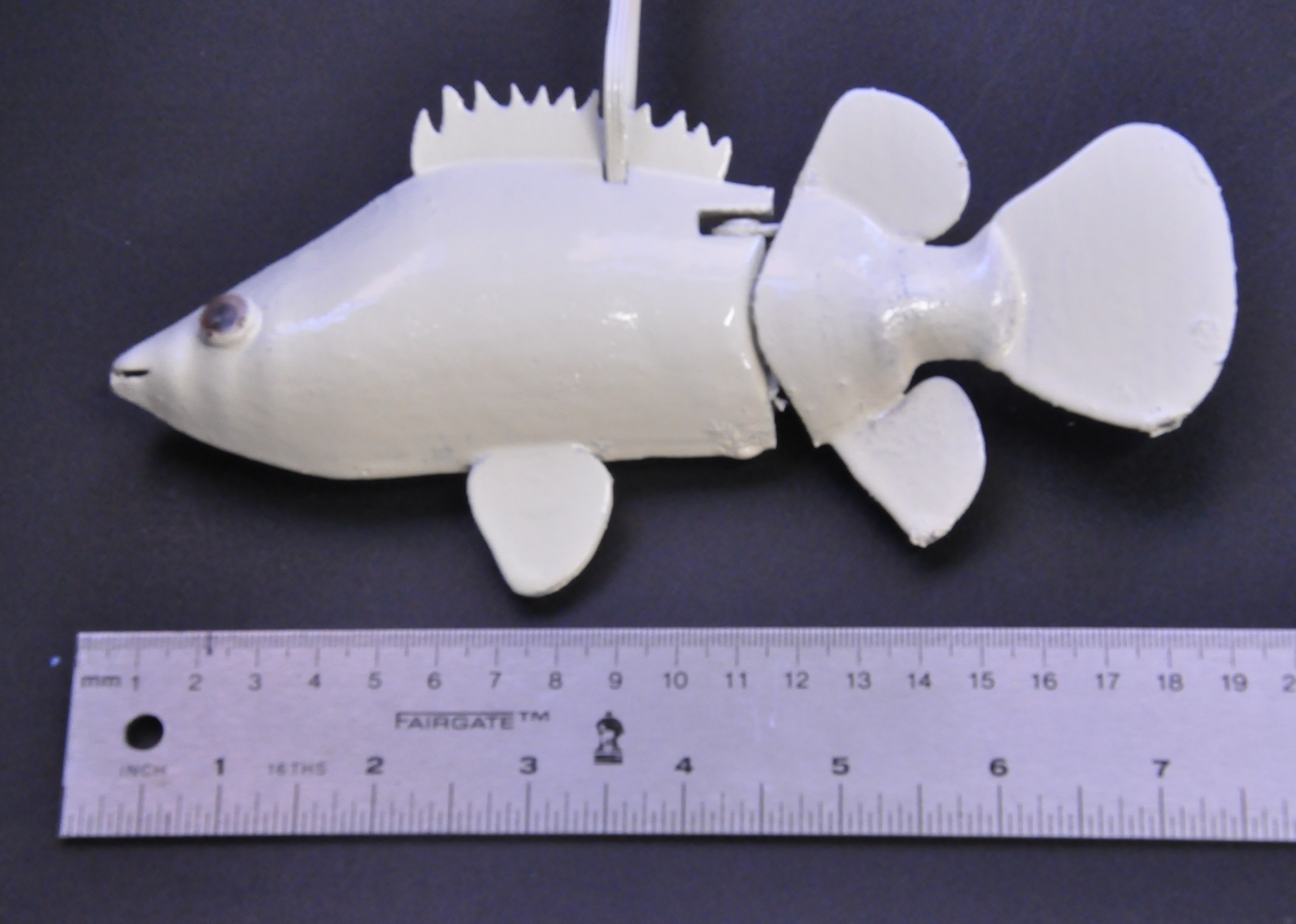

Supplement: Figure S4 — Illustration of the beige robot used in the control experiments investigating the effect of pigmentation. (JPG) [file pone.0069661.s004.jpg]

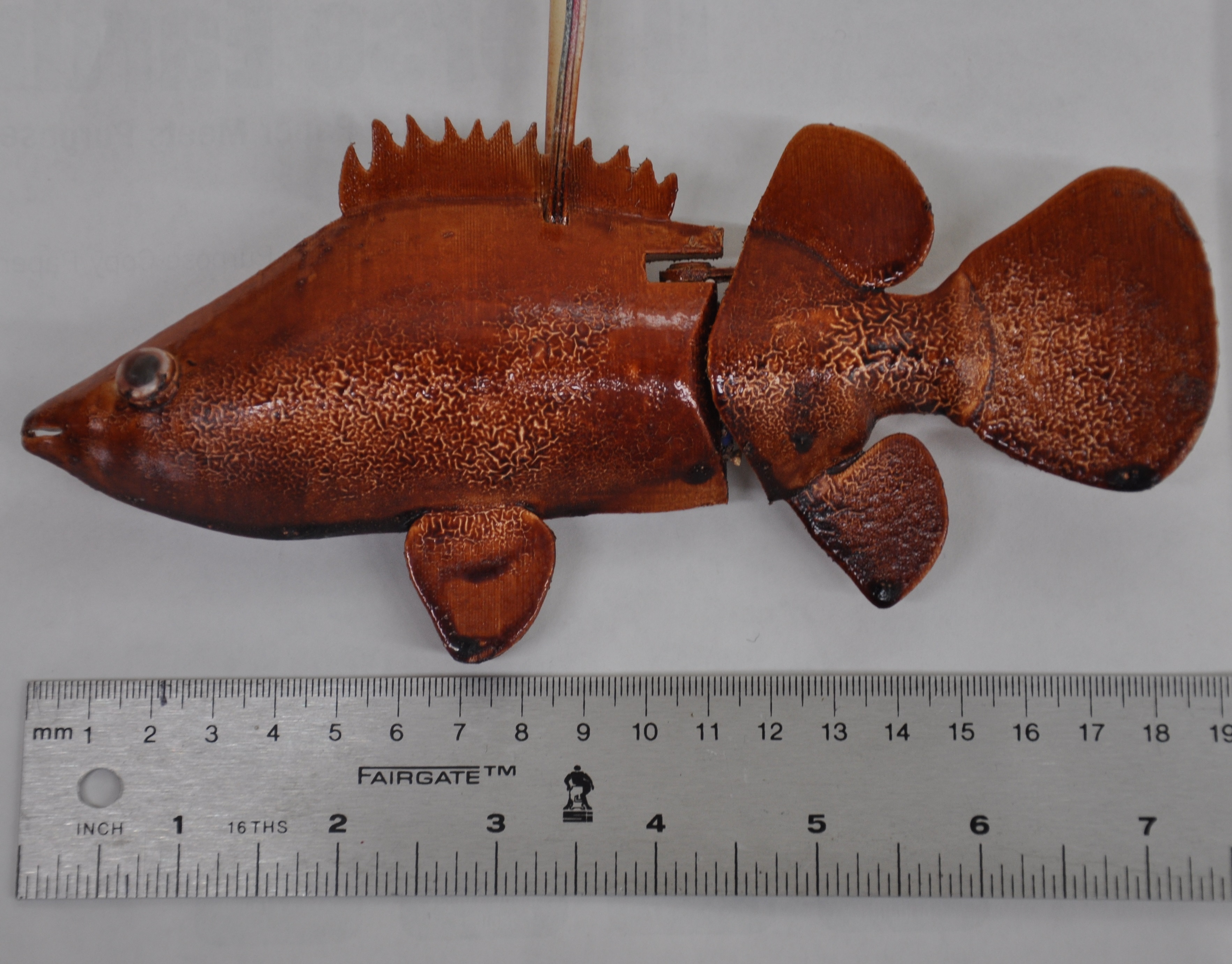

Supplement: Figure S5 — Illustration of the brown robot used in the control experiments investigating the effect of pigmentation. (JPG) [file pone.0069661.s005.jpg]
